# Screening fathers for postpartum depression in a maternal-child health clinic: a program evaluation in a midwest urban academic medical center

**DOI:** 10.1186/s12884-023-05966-y

**Published:** 2023-09-19

**Authors:** Sam Wainwright, Rachel Caskey, Aida Rodriguez, Abigail Holicky, Melissa Wagner-Schuman, Anne Elizabeth Glassgow

**Affiliations:** 1 Division of Academic Internal Medicine, College of Medicine, University of Illinois at Chicago, 820 S. Wood Street, CSN 440, M/C 718, Chicago, IL 60612 USA; 2 Department of Pediatrics, College of Medicine, University of Illinois at Chicago, Chicago, IL USA; 3 School of Public Health, University of Illinois at Chicago, Chicago, IL USA; 4 Department of Psychiatry and Behavioral Neuroscience, University of Cincinnati, Cincinnati, OH USA; 5 Department of Psychiatry, University of Illinois Chicago College of Medicine, Chicago, IL USA

**Keywords:** Fathers, Postpartum depression, Perinatal mental health, Paternal mental health, Screening

## Abstract

**Background:**

Postpartum depression (PPD) impacts fathers as well as mothers, and is estimated to affect between 8 and 13% of fathers. Paternal PPD is a risk factor for worsened quality of life, poor physical and mental health, and developmental and relational harms in the father-mother-child triad. There are no current recommendations for PPD screening among fathers. Paternal PPD screening was piloted in an intergenerational postpartum primary care clinic.

**Methods:**

The pilot was carried out in an intergenerational postpartum primary care clinic located at a Midwest urban academic safety net health system from October 2021 to July 2022. Fathers actively involved in relationships with mothers or infants receiving primary care in the clinic were approached with mothers’ permission. A novel survey instrument was used to collect demographic/social data, as well as mental health history and current stress levels; an Edinburgh Postnatal Depression Scale (EPDS) was also administered. Screenings were completed by social workers; data were collected in REDCap and descriptive statistics were calculated in SAS.

**Results:**

29 fathers were contacted and 24 completed screening (83%). Mean age was 31 years (range 19–48). Most (87%) identified as belonging to a racial or ethnic minority group. Fathers self-reported low rates of stress and preexisting mental health conditions, but 30% screened positive for PPD on EPDS (score of ≥ 8, or suicidal ideation). Gaps in health care were found, as one-quarter (26%) of fathers were uninsured and half (54%) did not have a primary care provider. After screening, two requested mental health services, and three established new primary care with a physician.

**Conclusions:**

Participation was high in a PPD screening pilot for fathers in a primary care setting. This small sample of fathers demonstrated significant peripartum mental health challenges unlikely to have been identified otherwise. For some participants, engaging in PPD screening was an effective tool to prompt their subsequent engagement with general health care. This pilot is a step toward incorporating the health of fathers into models for supporting the health of families. Expanding screening for paternal PPD into routine primary care is necessary to reach more affected fathers.

## Background

Postpartum depression (PPD) impacts fathers as well as mothers. Paternal PPD is thought to affect approximately 8 to 13% of fathers, and the prevalence can increase to 50% of fathers when the mother is also experiencing PPD [1–3]. Fathers’ experiences of PPD are often characterized by an insidious onset over a year and may peak in prevalence between three and six months postpartum [4]. Fathers may more frequently endorse irritability, indecisiveness, and restricted range of emotions compared to mothers [5–7]. A variety of risk factors have been identified for fathers developing PPD [8–10], including a history of mental illness [11], maternal PPD, relationship discord [12–14], poverty and unemployment [15], low educational attainment, unintended pregnancy [16], and sleep disruption [17]. Though diagnosis and treatment of PPD is the same in fathers and mothers, a universal screening system for fathers does not yet exist [18]. Fathers suffering from PPD are rarely identified in routine postpartum or pediatric care [19]. Fathers can feel that their experiences and needs are marginalized in the postpartum period, and can also express ambivalence or resistance to seeking support [6, 20].

Despite the well-recognized link between maternal health and newborn health, research and clinical practice continues to lag in addressing the interconnectedness of fathers and male partners in the health of the family unit [21, 22]. The American Academy of Pediatrics has increased emphasis on engaging and supporting fathers in the health of children over the last 20 years, reflecting the growing body of research on the influence of fathers on the health and development of their children [23, 24]. Early work has been done to integrate screening opportunities for fathers into pediatric visits, as well as into prenatal visits [25, 26]. However, there continues to be little integration of fathers into routine postpartum care, where paternal PPD could be identified.

Identifying and ameliorating PPD is a worthwhile perinatal health goal for fathers and families. Treating PPD can lead to improved quality of life and improved relationships between parents and with the child [27, 28]. Fathers without depression can have a protective effect on the children of mothers suffering from depression, and conversely co-occurring paternal depression can worsen the severity of maternal PPD [29, 30]. Identifying and addressing paternal PPD may be an important and underutilized avenue for improving parental and familial mental health [7, 31]. Reviews of various screening tools to detect PPD in fathers have found that the Edinburgh Postnatal Depression Scale (EPDS), commonly used to screen for PPD in mothers, is a valid and reliable instrument for fathers [32–35]. It has been validated across a wide array of time-points from the antepartum period out to 12 months postpartum [35].

During newborn care visits at the University of Illinois at Chicago Health System (UI Health), health care providers observed that many fathers attending their child’s pediatrician visits seemed to be struggling with symptoms of postpartum stress, anxiety, and depression. In response, the UI Health Two-Generation Clinic expanded an existing maternal PPD screening program to include fathers in October 2021. A program evaluation was performed in November 2022 to evaluate the screening of new fathers for PPD during routine maternal postpartum and newborn pediatric care.

## Methods

### Clinic setting

The program evaluation was conducted in a primary care clinic located within an academic medical center where pediatric and maternal health care are provided in the same setting (UI Health Two-Generation (Two-Gen) Clinic). The aim of Two-Gen is to deliver integrated, comprehensive postpartum primary care to mothers and their children. The clinic is housed within the combined internal medicine and pediatrics (med-peds) clinics at UI Health. The clinic predominately serves a publicly-insured population of economically marginalized, families of color who reside on the South and West Sides of Chicago, Illinois. Two-Gen was designed to systemically address disparities by offering a model of population health that provides comprehensive screening and services to address the health, behavioral health, and social service needs of postpartum mothers and children. The clinic has a collaborative care model that utilizes a transdisciplinary team of primary care physicians, a psychiatrist, social workers, lactation consultants, and care navigators/health coaches. Dyad clinic visits are co-scheduled (at the same time), co-located (in the same exam room), with the same primary care provider.

### Sample

During the screening period from October 2021 through July 2022 there were approximately 60 mother-infant dyads receiving care in the Two-Gen clinic. We sought to identify all fathers actively involved in a relationship with a patient (mother or infant) in the clinic. Our intention was to be inclusive of biological and non-biological fathers, though only biological fathers were identified during the screening period. We contacted all mothers for whom there was not known intimate partner violence or other safety concern and asked them to identify their child’s father and for their permission to contact him.

### Procedures

A mother-as-gatekeeper model was utilized to minimize unintended and unwanted consequences of contacting fathers, including privacy concerns and potential exacerbation of partner conflict (e.g., intimate partner violence). After receiving the mother’s permission to contact fathers, clinic staff (a female social worker, a male social work master’s student, or a female care navigator) contacted fathers either by phone, text message, or during an in-person clinic visit to explain the screening process, obtain permission to participate, and to administer the screening. Fathers were informed that their information was confidential and would not be shared with their partners unless the father requested it be shared. Providers administered the screening at the clinic visit (in the waiting area, in a separate room, or in the room with the mother/baby if preferred) or by phone. If in-person, interviewers presented fathers with the option of independently completing the screening on a tablet or responding to the questions verbally.

Following the screening, fathers were provided with mental health education and resources, and offered referrals to medical and/or mental health care, regardless of screening results. Clinic social workers met with all fathers with a positive screen or a safety concern immediately following the screening to provide mental health referrals, including assistance in establishing care.

### Measures

Demographic and social data were collected including age, race/ethnicity, insurance status, marital status, cohabitation, and enrollment with a primary care provider. Mental health history included three yes/no questions about ever receiving a diagnosis, medication, and/or counseling for a mental health problem. Fathers were asked about stress in three domains (financial, relationship with partner, and role as new father) on a scale of one to five, with five being the most stressful. Depression symptoms were measured using the 10-item self-rated EPDS questionnaire [32]. The EPDS is a reliable and validated measure for mood symptoms in fathers [33–35]. Each item has four short statements/responses about common depressive symptoms experienced over the past seven days. The questions are scored from 0 to 3, with at total range of questionnaire scores from 0 to 30. A higher score indicates a higher level of depression symptoms. For mothers, a cutoff score of ≥ 10 indicates possible depression and is considered a positive screen. For fathers, previous studies suggest using lower cutoff scores ranging from 7 to 10 [34, 35]. For our analysis, ≥ 8 and/or any endorsement of question #10 (thoughts of self-harm) was considered a positive screening. The design of the screening was not qualitative; however, summative comments and quotes from fathers were recorded contemporaneously by the social workers. Authors interviewed the social workers after the pilot to gather their impressions. This information is reported purely for context.

### Analysis

Descriptive statistics (frequencies for categorical measures; means and standard deviations [S.D.] for continuous measures) were computed for sample demographic/social characteristics, mental health history, stress, and EPDS. Data were analyzed using Microsoft Excel and SAS 9.4 statistical software package. The project was approved by the University of Illinois at Chicago Institutional Review Board with a waiver of informed consent under protocol #2022 − 1621 as a clinical quality improvement program.

## Results

We received permission to contact twenty-nine fathers, of whom twenty-four (83%) completed the screening. The five fathers who did not complete the screening did not respond to phone calls and text messages requesting their participation. The timing of completed screenings varied among fathers, with an average screening time at seven months postpartum (range: 1–15 months). Table 1 summarizes the sample characteristics. Fathers’ average age was 31 years old (range 19–48). The sample was diverse with 54% identifying as non-Hispanic Black, 33% as Hispanic and 13% as another racial group. Most fathers had insurance (74%), yet of note, more than half (54%) did not have a primary care provider. Most fathers were married/partnered (75%) and were living with their partner and child (86%).

**Table 1:** Characteristics of Fathers Screened^ for Postpartum Depression (n = 24)

Characteristic	Count (%*)
Age	
Younger than 30 Years	10 (45%)
30 Years and Older	12 (55%)
*Missing*	2
Race/Ethnicity	
Non-Hispanic Black	13 (54%)
Hispanic	8 (33%)
Non-Hispanic Other (includes White)	3 (13%)
Insurance Status	
Private	9 (39%)
Public (includes Veterans Affairs)	8 (35%)
Uninsured	6 (26%)
*Missing*	1
Marital Status	
Married/Partnered	18 (75%)
Separated/Divorced	6 (25%)
Lives with Partner and Child	
Yes	19 (86%)
No	3 (14%)
*Missing*	2
Primary Care Provider	
Yes	11 (46%)
No	13 (54%)

^Screened between October 2021 and July 2022

*Respondents with missing data were not included in reported percentages

**Fig. 1:**
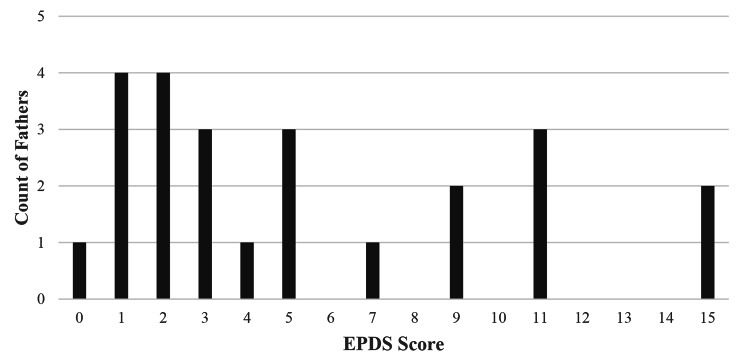
Distribution of Edinburgh Postnatal Depression Scale scores among fathers screened (n = 24)

Fathers self-reported low rates of past mental health diagnosis and/or treatment (Table 2). Only two (8%) reported being diagnosed with a mental health disorder and one (4%) reported use of medication, whereas five (22%) reported seeing a counselor for mental health problems in the past. Fathers reported overall low levels of stress across all three domains (i.e., money, relationship, and role as new father). Fathers’ EPDS scores ranged from 0 to 15, in a bimodal distribution (Fig. 1) with a mean of 5.3 (S.D.= 4.6). Approximately one third of fathers (30%) screened positive for PPD based on our pre-specified criteria (score ≥ 8 and/or endorsement of any suicide ideation). One father reported thoughts of harming himself.

**Table 2:** Mental Health History, Stress Level and Depression Screening among Fathers Screened (n = 24)

Screening Question	
**Mental Health History**	Yes Count (%*)
Have you ever been diagnosed with a mental health problem such as anxiety or depression?	2 (8%)
Have you ever taken medication for a mental health problem such as anxiety or depression?	1 (4%) *Missing = 1*
Have you ever seen a counselor for a mental health problem such as anxiety or depression?	5 (22%) *Missing = 1*
**Stress During the Past Month^**	**Mean Score (S.D.)**
How stressed have you been about money?	2.2 (1.3)
How stressed have you been about your relationship with your significant other?	1.5 (0.9)
How stressed have you been about your new role as a father?	1.4 (0.9)
**Edinburgh Postnatal Depression Scale**	**Positive** ^**#**^ **Count (%)**
	7 (30%)
	**Mean Score (S.D.)**
	5.3 (4.6)

*Respondents with missing data were not included in reported percentages

^ Scale of 1–5, with 1 being least stressed and 5 being most stressed

^#^ Positive is EPDS score of eight or higher or an endorsement of item 10 (the thought of harming myself has occurred to me)

All fathers were offered mental health educational materials, mental health services, and connection with a primary care provider. Of the 24 fathers screened, two requested mental health services, and three established new primary care with a physician.

### Qualitative participant comments

Social workers were interviewed by the authors after the screenings were completed, to gather their impressions of their conversations with fathers. Social workers noted fathers expressed having active roles in their infant’s lives and prioritized the health of their infants and partners over their own. Some wanted to address their own mental health, and wanted mental health and community resources. Many fathers expressed significant symptoms of lack of sleep, frequently citing fatigue as a driver of psychosocial distress.

Many of the fathers interviewed were young, first-time parents and expressed to the social workers fears of “not doing things right” and not knowing how to participate in the many mundane tasks of parenting an infant such as holding, diapering, bottle-feeding, and soothing. Fathers often identified strongly with the “provider” role and expressed sentiments that early infant care was a “mother’s job.” They were simultaneously eager to support the mother of their child – particularly when perceiving signs of maternal PPD – but found the demands of working and providing economic support to conflict with increased needs to support mother and baby.

Despite low scores in many areas of the screening instrument, fathers did acknowledge significant stress, anxiety, and even depression themselves, but were often resistant to receiving direct support. The social workers felt that many fathers were more receptive to receiving resources when it was characterized as routine “for everyone,” rather than responsive to a perceived personal diagnosis or deficit.

## Discussion

In our small sample of fathers, 7 of the 24 (30%) who completed the survey screened positive for PPD. This rate is higher than typically reported in the general population of fathers (8–13%) [1]. The high rate of a positive PPD screening comes despite a low rate of self-reported background mental health diagnoses in our cohort. This aligns with concerns that PPD is a significantly underdiagnosed disease among fathers [36]. We suspect this high rate reflects the population of socially vulnerable families served by the Two-Gen clinic, burdened by many of the social determinants of health – poverty, housing instability and segregation, violence, lack of family and social support – that are known risk factors for postpartum depression [10]. Additionally, 31.5% of mothers receiving care in Two-Gen have screened positive for depression or anxiety at least once during their care [unpublished data], aligning our findings with past work that has highlighting maternal PPD as a risk factor for paternal PPD [28].

Prior research has found that fathers can feel marginalized or excluded from perinatal care spaces and can also be resistant to interventions conceived to foster their inclusion. This may be driven by many factors including lack of paid parental leave, inconvenient scheduling during work hours, social biases away from men taking time off to take their children to the doctor, and negative and excluding experiences in maternal-child care spaces including the delivery room [37–40]. In our experience, however, fathers were accepting of PPD screening and were pleasantly surprised to have the focus on them, their perceptions, and needs. This is confirmed by the high rate of participation in the screening and the experience of the social workers interviewing the fathers. Many mothers also were supportive of the fathers being screened and expressed a perception of their partner having unmet needs. About half the fathers did not have regular attachment to primary care and almost a quarter reported being uninsured. This suggests additional avenues (primary care connection, insurance enrollment navigation) for engaging fathers in PPD screening and other health promoting activities for themselves.

### Limitations

This program evaluation had multiple limitations. It is a retrospective review of a structured clinical screening intervention, and thus may not fully capture the complex nature of PPD in fathers. The intervention was designed to understand fathers’ willingness to participate in screening and their broader peripartum mental health needs; it was not designed to make a clinical diagnosis of PPD. Our decision to use the lower EPDS cut-off score for fathers increases the risk for over-identification of PPD. Conversely, the wide range of screening timepoints (1 to 15 months postpartum) raises the possibility of under detection of PPD, as some research suggests peak prevalence of paternal PPD around three to six months postpartum, though this data is limited [18, 29].

We were intentional about which fathers were contacted and acknowledge that the mother-as-gatekeeper approach introduces bias into our sample. Fathers where there was a concern or history of abusive, threatening, or dangerous behavior were not contacted. This decision reflects significant concerns about maternal and child safety regarding IPV which are often heightened in the pregnancy and postpartum period [41, 42]. However, fathers who commit IPV may be particularly high-risk in terms of comorbid mental health needs and trauma, and may represent an important, sizeable, though challenging sub-group for which to develop effective screening, treatment, and engagement strategies.

86% of our fathers reported cohabitation with the child’s mother, indicating a shared milieu and potentially strong inter-family effects and needs. However, this high rate of cohabitation may reflect the approach we used, mother-as-gatekeeper, for accessing fathers to screen. A more universal approach to screening might find different results as non-residential fathers may have different needs and experiences.

## Conclusions

Our findings suggest that fathers are willing to participate in PPD screening, though they may be less willing to engage directly with mental health services. We found that in our small sample there was higher than expected rates of positive screening for paternal PPD, and resistance to engaging with individual mental health services among those who screened positive. By welcoming fathers as an integral part of the health (and health care) of the family, our efforts acknowledge the ways in which expanding the focus of maternal-child health care teams can contribute to the improved functioning and optimal health of the entire family-unit. Additional research is needed to identify the optimal times and venues for paternal engagement, including exploring the preferences of fathers themselves.

## Data Availability

The dataset generated and analysis for this study are not publicly available due patient privacy but are available from the corresponding author on reasonable request.

## Abbreviations

EPDS: Edinburgh Postnatal Depression Scale
PPD: Postpartum depression
S.D: Standard deviations
UI Health: University of Illinois at Chicago Health System
